# Geriatric Respondents and Non-Respondents to Probiotic Intervention Can be Differentiated by Inherent Gut Microbiome Composition

**DOI:** 10.3389/fmicb.2015.00944

**Published:** 2015-09-08

**Authors:** Suja Senan, Jashbhai B. Prajapati, Chaitanya G. Joshi, V. Sreeja, Manisha K. Gohel, Sunil Trivedi, Rupal M. Patel, Himanshu Pandya, Uday Shankar Singh, Ajay Phatak, Hasmukh A. Patel

**Affiliations:** ^1^Department of Dairy Science, South Dakota State University, Brookings, SD, USA; ^2^Department of Dairy Microbiology, Anand Agricultural University, Anand, India; ^3^Department of Animal Biotechnology, Anand Agricultural University, Anand, India; ^4^Department of Community Medicine, H. M Patel Center for Medical Care and Education, Karamsad, India; ^5^Department of Microbiology, H. M Patel Center for Medical Care and Education, Karamsad, India; ^6^Department of Medicine, H. M Patel Center for Medical Care and Education, Karamsad, India; ^7^Central Research Services, Charutar Arogya Mandal, Karamsad, India

**Keywords:** geriatric, gut, metagenome, probiotics, MTCC 5463

## Abstract

**Scope:**

Probiotic interventions are known to have been shown to influence the composition of the intestinal microbiota in geriatrics. The growing concern is the apparent variation in response to identical strain dosage among human volunteers. One factor that governs this variation is the host gut microbiome. In this study, we attempted to define a core gut metagenome, which could act as a predisposition signature marker of inherent bacterial community that can help predict the success of a probiotic intervention.

**Methods and results:**

To characterize the geriatric gut microbiome, we designed primers targeting the 16S rRNA hypervariable region V2–V3 followed by semiconductor sequencing using Ion Torrent PGM. Among respondents and non-respondents, the chief genera of phylum Firmicutes that showed significant differences are *Lactobacillus*, *Clostridium*, *Eubacterium*, and *Blautia* (*q* < 0.002), while in the genera of phylum Proteobacteria included *Shigella*, *Escherichia*, *Burkholderia* and *Camphylobacter* (*q* < 0.002).

**Conclusion:**

We have identified potential microbial biomarkers and taxonomic patterns that correlate with a positive response to probiotic intervention in geriatric volunteers. Future work with larger cohorts of geriatrics with diverse dietary influences could reveal the potential of the signature patterns of microbiota for personalized nutrition.

## Introduction

An integrative study of the host and its surrounding environment is imperative to comprehend the complex biological system of the human body. As a part of the environment, the human host more than 100 trillion bacteria forming the “in-vironment” (de Wouters et al., 2000) made up of millions of microbial genes in the intestine (Lederberg, 2000). The indigenous microbial community plays an integral role in regulating the host’s physiological, nutritional, and immunological processes (Hooper et al., 2001). The study of the diversity of the indigenous microbial community can explain the host microbe interaction (Gerritsen et al., 2011). The gut microbiota composition changes with age due to physiological reasons and increased use of medications (Bartosch et al., 2004; Mueller et al., 2006; Mariat et al., 2009; Zwielehner et al., 2009). The microbiota of elderly people showed a higher *Bacteroidetes*/*Firmicutes* ratio along with a high inter-individual variation in microbiota composition at the phylum level when compared with young adults (Claesson et al., 2011). Studies of the geriatric gut have shown a decline in the count and diversity among Bacteroidetes (Hopkins and Macfarlane, 2002; Woodmansey et al., 2004; Guigoz et al., 2008). Proteolytic bacteria increase on aging in the large bowel, leading to putrefaction (Hopkins et al., 2001). Such changes in intestinal microbiome cause prolonged intestinal transit time and fecal retention among geriatrics (Tiihonen et al., 2009). An understanding of the changes in the microbiome of the elderly has led to the possibility of correcting the dysbiosis by administering probiotics. Probiotics have been successful in increasing the levels of health-promoting bacteria in the fecal microbiota of elderly (Ahmed et al., 2007; Lahtinen et al., 2009; Matsumoto et al., 2009), improving the frequency of bowel movements (Pitkala et al., 2007), *Clostridium difficile*-associated diarrhea incidence (Ouwehand et al., 2009), and frequency of defecation (An et al., 2010).

The translation of the above-mentioned benefits of probiotics to the host cannot be guaranteed. This could be due to the individual differences with respect to diet, the structure and operations of the gut microbiota, nutrient and energy harvest, variations in human environmental exposures, microbial ecology, and genotype (Turnbaugh et al., 2009). In order to assure uniform outcomes of therapy among subjects, the International Scientific Association for Probiotics and Prebiotics (ISAPP) had come up with recommendations for conducting a well-defined trial (Reid et al., 2010). Briefly, they include (1) clearly define the end goal, (2) design the study by identifying precise parameters and defining the level of response that will be tested, (3) base the selection of the intervention on scientific investigations, and (4) carefully select the study cohort. Inter-individual diversity in responses toward probiotics could also be due to core gut microbiome patterns. Recently, role of microbial biomarkers for determining dietary responsiveness were identified in obese individuals (Korpela et al., 2014) and metabolic diseases (McOrist et al., 2011; Walker et al., 2011; Louis, 2012; Lampe et al., 2013), paving the way for personalized nutrition. This study takes up the challenge to identify the factors that differentiate a respondent from a non-respondent and utilize the findings to define the precise dose and response prognosis. This finding can help design probiotic supplements catering to a niche market defined by age, location, or disease state.

From an Indian perspective, gut metagenomics have been studied in malnourished children (Gupta et al., 2011), obese individuals (Patil et al., 2012), and children of varying nutritional status (Ghosh et al., 2014). It was for the first time in India that the present study was conducted to investigate the elderly gut metagenome to identify microbial biomarkers determining responsiveness of the host to a probiotic therapy. We hypothesized that by studying the baseline gut microbiota diversity of elderly subjects, we could identify a core gut microbiome signature pattern that is likely to positively influence the response of an individual to the probiotic strain. The strain under study, *Lactobacillus helveticus* MTCC 5463 is an indigenous potential probiotic with *in vitro*, *in vivo*, and *in silico* studies providing suggestive evidences of the strain’s robustness in the gut and transit, adhesion, autoaggregation, colonization, antibacterial property, hypocholesterolemic, and immunomodulatory properties (Senan et al., 2015). The outcome of this study paves the way forward for tailored probiotic therapy.

## Materials and Methods

### Origin and maintenance of bacterial strains

The indigenous probiotic strain *L. helveticus* MTCC 5463 (Prajapati et al., 2011) and starter culture *Streptococcus thermophilus* MTCC 5460 (Prajapati et al., 2013) were maintained by the Department of Dairy Microbiology, Anand Agricultural University, India at −80°C as 15% glycerol stocks and were routinely cultured in de Man, Rogosa, Sharpe (MRS) and M 17 medium, respectively (HiMedia India Ltd., India).

### Product preparation

The test product was a fermented probiotic drink (*Lassi*) with double toned milk fermented with culture containing *S. thermophilus* MTCC 5460 and *L. helveticus* MTCC 5463. The cultures were added at 0.1% each and incubated aerobically till an acidity of 0.8–0.9% lactic acid was obtained. Both the test and placebo products contained sugar and prebiotic honey in a standardized ratio. The control product was made in a similar manner without the addition of MTCC 5463. The shelf life of the fermented drink was 28 days at 4°C, corresponding to the lower level (10^9^ CFU/ml) of strain MTCC 5463.

### Participant selection

Individuals ranging from 64 to 74 years were recruited. Initially, 112 subjects were enrolled in the trial, 36 had to withdraw because of antibiotic consumption. Volunteers were asked to sign the consent form before recruitment. Exclusion criteria included lactose intolerance, recent antibiotic treatment, frequent gastrointestinal disorders, or metabolic diseases. Participants included in the trial had no known allergies or intolerance to dairy foods. The trial had 80% power at a 5% 2-sided significance level to detect a >50% change in the primary outcome among subjects. No antibiotics or laxatives were taken 2 months before or during the study.

### Intervention

Sixteen participants showing diversity in lactobacilli count and cholesterol levels were involved in the double-blind, crossover, placebo-controlled, and randomized-feeding trial. The trial was divided into five consecutive periods: a pre-feeding period (2 weeks), followed by a feeding period (4 weeks), a washout period (4 weeks), a second-feeding period (4 weeks), and a final washout period (2 weeks).

### Collection and analysis of the blood samples

Blood samples were taken from each volunteer immediately before and after each treatment period using EDTA-containing vacutainers. Total cholesterol (TC) was measured using enzyme-spectrophotometry kits and IgG, IgM, TNF-alpha, INF-gamma, and IL-2 by ELISA capture assay (Siemens Medical Solutions Diagnostics Ltd., India). All the tests were done at the Central Diagnostic Laboratory, Shri Krishna Medical College Karamsad, Anand, Gujarat, an NABL accredited and ISO 15189:2003 laboratory.

### Selection of respondents and non-respondents

A respondent was defined as a subject having an improvement in the levels of *L. helveticus* MTCC 5463 strain count in feces and a reduction in cholesterol levels. Similarly, a non-responder was defined as a subject who displayed an absence of decrease in cholesterol levels and increase in viability of the bacterial strain. Based on these criteria, we identified eight subjects each in respondents and non-respondents category.

### Fecal sample collection

Single fecal samples were collected at the end of every 2 weeks. Participants were given 60 ml sterile stool container with a sterile plastic spoon (Polylab Plasticware, India) and were asked to fill the tube to the 30 ml mark with feces from the midstream defecation period. During the second-feeding period, there was a crossover of the feeding design. For every collection, the stool samples were immediately frozen at −20°C.

### DNA extraction from stool samples

DNA was extracted from feces using a QIAamp MiniPrep DNA extraction kit following the manufacturer’s instructions. The DNA was stored at −20°C. Quality and purity of the isolated genomic DNA were confirmed by agarose gel electrophoresis and spectrophotometry on the NanoDrop 2000 device (Fisher Scientific, Schwerte, Germany). DNA concentration was estimated with the Qubit 2.0 instruments applying the Qubit dsDNA HS Assay (Life Technologies, Invitrogen division, Darmstadt, Germany).

### Quantification of lactobacilli in stool

The subjects voided their feces into a 60-ml sterile stool container with a sterile plastic spoon (Polylab Plasticware, India) from the midstream defecation period. Within an hour of sample procurement, samples were diluted and homogenized to give a 10-fold dilution (wet weight/volume). Stool Lactobacilli content was determined by plating aliquots (1 ml) of each dilution on freshly prepared de Mann Rogosa Sharp agar (Himedia, India), incubated for 24–48 h at 37°C under anaerobic conditions. Stool *L. helveticus* MTCC 5463 content was determined by quantitative PCR (qPCR) using StepOne Real-Time PCR System (ABI/Thermo Fisher Scientific, Bangalore, India). Primers and 3′ minor groove binder (MGB) probes for accurate detection and quantification of *L. helveticus* MTCC 5463 in human fecal samples were developed with Primer Express v3.0 [Thermo Fisher Scientific (earlier Applied Biosystems), Bangalore, India]. The temperature profile of the qPCR consisted 2 min at 50°C, 10 min at 95°C, followed by 45 cycles of 15 s at 95°C, and 1 min at 60°C. Species specific primers and probe targeted on the bile salt hydrolase gene of *L. helveticus* MTCC 5463 (Accession number AEYL01000315; locus tag AAULH_13111 2049 bp). BLAST^1^ and EMBL^2^ database were used to ensure the specificity of the primers. Genomic DNA standards prepared with six different serial dilutions (2.68 × 10^6^, 2.68 × 10^5^, 2.68 × 10^4^, 2.68 × 10^3^, 2.68 × 10^2^, and 2.68 × 10) being equivalent to ranges from 10^6^ to 10^1^ CFU/ml of target genome (MTCC 5463). The cycle threshold (*C*_T_) was evaluated to create the standard curve. The amplification efficiencies were determined using the formula *E* = [10^(−1/slope)^ − 1].

### 16S primers and amplicon library generation

PCR amplification of the 16S rRNA hypervariable region V2–V3 was performed with a pool of 32 degenerated forward and 1 degenerated reverse primer targeting bacteria as described by Schmalenberger et al. (2001) with barcode sequences at the 5′ end of the primers. Primers were assessed for specificity using the SILVA 108 SSU Reference 16S rRNA gene database and BLASTN matches with corresponding 16S rRNA gene sequences. The addition of the barcodes to the primers resulted in an amplicon approximately 430 nt in length. The primers were diluted and pooled to equimolar quantities. For amplicon library preparation, 4 ng of each genomic DNA, 5 mM dNTPs, 2 mM MgCl_2_ (Roche Diagnostics, USA), 1 U Platinum Taq DNA polymerase High Fidelity, and 10 pmol primer-mix were used per 25 μl amplification reaction. The PCR conditions were as follows: 95°C for 5 min, followed by 30 cycles of 94°C for 15 s, 60°C for 45 s, 70°C for 30 s, and a final elongation step of 72°C for 10 min. Amplicon product purification was performed via gel electrophoresis on a 1.5% Tris Borat EDTA agarose gel-stained with ethidium bromide (EtBr) (Life Technologies). All positive PCR reactions were electrophoresized in agarose gels and products with the expected size were cut and purified with Qiagen Gel extraction kit (Qiagen, Düsseldorf, Germany). The exact fragment sizes were determined using HT DNA High Sensitivity LabChip Kit (Caliper Life Sciences GmbH, Mainz, Germany). Amplicon library concentration was estimated with the Qubit 2.0 instrument using the Qubit dsDNA HS assay (Life Technologies).

### Emulsion PCR and sequencing

The emulsion PCR was carried out applying the Ion XPress Template kit V2.0 (Life Technologies) as described in the appropriate user Guide (Part No. 4469004 Rev. B 07/2011) provided by the manufacturer. Quality and quantity of the enriched spheres were checked on the Guava easyCyte5 system (Millipore GmbH, Schwalbach am Taunus, Germany) as described in the appendix of the Ion Xpress Template Kit User Guide (Part Number 4467389 Rev. B, 05/2011). Sequencing of the amplicon libraries was carried out on the Ion Torrent Personal Genome Machine (PGM) system using the Ion Sequencing 200 kit (all Life Technologies) following the corresponding protocol (Part No. 4471999 Rev B, October 13, 2011). Quality check passed libraries were subjected to emulsion PCR using the Ion PGM 200 Xpress Template Kit (Life Technologies). After bead enrichment, beads were loaded onto Ion 316 chips and sequenced using an Ion Torrent PGM.

### Sequence analysis

The sequence data sets obtained were uploaded to the Metagenome Rapid Annotation using Subsystem Technology (MG-RAST) server^3^ and subsequently checked for low-quality reads. The sequence reads that passed the quality filtering step were then subjected to further analysis of the taxonomic annotation of the fecal DNA sequences using QIIME pipeline (Caporaso et al., 2010). To investigate the species diversity, we used rarefaction curves, and richness estimators like Chao1 in QIIME. Statistic comparison of samples organized into respondents and non-respondents for the deferentially abundant microbial diversity was studied using an alignment platform, STAMP.^4^

## Results

We conducted a comparative analysis of respondents and non-respondents fecal microbiome to reveal differences and identified biomarkers that differentiate them.

### Quantification of lactobacilli count using traditional plating and qPCR

Traditional plate counts of lactobacilli at genus level on selective medium ranged from a baseline reading of 8.6 log CFU/g of wet fecal matter, which rose to 9.3 log CFU/g at the end of feeding period and a gradual decrease to 8.7 log CFU/g at the end of the placebo feeding. The qPCR primers targeted the bile salt hydrolase gene of MTCC 5463, which made the gene copy count a fraction of the plate count. On the other hand, the precise *L. helveticus* MTCC 5463 strain count from real-time PCR showed a complete absence of the strain before feeding. At the end of 30 days, the strain appeared in the feces of all subjects in the treated group, reaching a level as high as 8.32 to the lowest amount of 6.17 log gene copies/g fecal matter at end of feeding period.

### Summary of sequence processing data

Primers targeting the 16S rRNA gene V2–V3 region (Schmalenberger et al., 2001) precisely generated amplicons from members of the domain bacteria and did not hybridize to sequences of the domains Archaea and Eucarya. By using the ARB SILVA 108 SSU database, a high match of 84.5% at a maximum number of four mismatches was observed. The primer pair theoretically targeted all 16S rRNA gene sequences of the gut microbiota bacterial orders and generated a single amplicon. All 32 amplicons from the pool of test and placebo groups were mixed together at an equimolar ratio. After pooling and elution of the amplicons from the gel, we got one band for the probiotic fed group of 520 bp having a concentration of 1912.09 pg/μl and molarity of 5562.5 pmol/l. The placebo fed group gave an amplicon of 513 bp having a concentration of 1435.87 pg/μl and 4237.7 pmol/l molarity. The data sets for 16 subjects before probiotic feeding had reads ranging from 13,061 to 980,628 with a read length ranging from 201 to 251 bp and a total amount of 42,52,62,470 bases.

### Inter-individual differences in shifts in phyla abundance (%) before and after probiotic feeding

**Figure 1:**
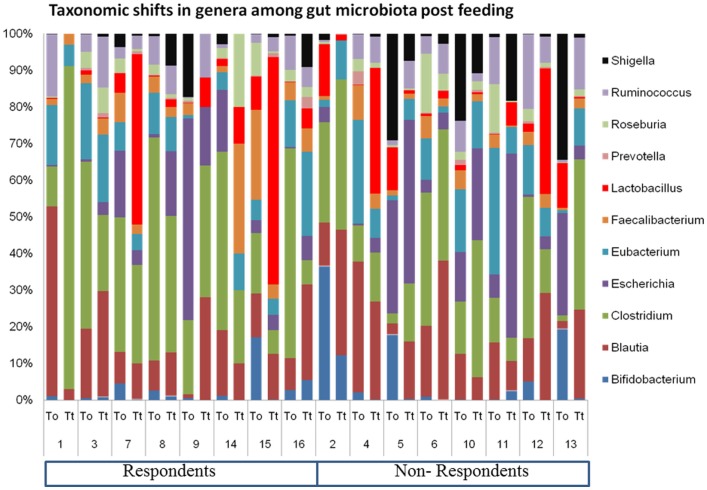
**Inter-individual variation in the geriatric gut microbiome pre- and post-probiotic feeding**.

The relative abundance of major genera in the elderly gut metagenome and high-level of inter-individual variation is shown in Figure 1. We presume that the inter-individual differences are indicative of a highly personal fecal microbiota profile, which determines the response of the host to probiotics. Host factors probably play a major effect in shaping the intestinal microbial ecosystem during an intervention. In the present study, we attempted to understand the core microbiome of respondents and non-respondents to probiotics.

### Probiotic feeding and effect on lipid profile and immunologic parameters

In addition to TC (primary outcome), in this study, we also investigated the effect of probiotic intervention on lipid profile, beta glucouronidase activity, and immunological parameters. The mean β-glucuronidase activity was reduced in test group from 1.40 to 0.73 (microgram/min/mg of protein), while in case of placebo group, no effect on enzyme activity was observed. A significant immunomodulatory effect on the TNF-α and IL-2 levels in subjects among probiotic group compared to placebo group was observed. There was however no significant beneficiary effect found on IFN-γ, IgG, or IgM levels. Paired *t*-test showed that there were statistically significant differences in serum cholesterol, VLDL, TC/HDL, LDL/HDL in placebo group and in LDL, TC/HDL, and LDL/HDL in probiotic group. A significant (*p* = 0.01) decrease in the LDL value was seen in the probiotic group at the end of 30 days of feeding (Table 1).

**Table 1:** **Effect of probiotic and placebo interventions on lipid profile in geriatric subjects**.

Variables	Probiotic (means ***±*** SD)	Placebo (means ***±*** SD)
**Total cholesterol (TC) (mg/dL)**
Baseline	161.67 ± 41.05	174.32 ± 49.99
Post-intervention	158.09 ± 42.63	167.09 ± 43.11
*p*-Value	0.12	<0.001
**Triglyceride (mg/dL)**
Baseline	103.77 ± 49.84	116.38 ± 71.01
Post-intervention	104.00 ± 56.43	108.58 ± 70.74
*p*-Value	0.96	0.03
**High density lipoprotein (HDL) (mg/dL)**
Baseline	46.21 ± 12.46	49.67 ± 15.97
Post-intervention	47.08 ± 13.97	48.77 ± 12.98
*p*-Value	0.24	0.34
**Low density lipoprotein (LDL) (mg/dL)**
Baseline	98.48 ± 37.12	88.93 ± 38.37
Post-intervention	92.93 ± 35.79	84.56 ± 31.13
*p*-Value	0.01	0.09
**Very low density lipoprotein (mg/dL)**
Baseline	21.63 ± 12.04	23.28 ± 14.20
Post-intervention	21.34 ± 11.97	21.71 ± 14.12
*p*-Value	0.74	0.03
**Total cholesterol/HDL (mg/dL)**
Baseline	3.91 ± 1.22	3.77 ± 1.33
Post-intervention	3.74 ± 1.20	3.65 ± 1.23
*p*-Value	<0.001	0.04
**LDL/HDL (mg/dL)**
Baseline	2.37 ± 0.96	2.23 ± 1.01
Post-intervention	2.21 ± 0.91	2.13 ± 0.96
*p*-Value	<0.001	0.04

### Participant diversification into respondents and non-respondents

**Figure 2:**
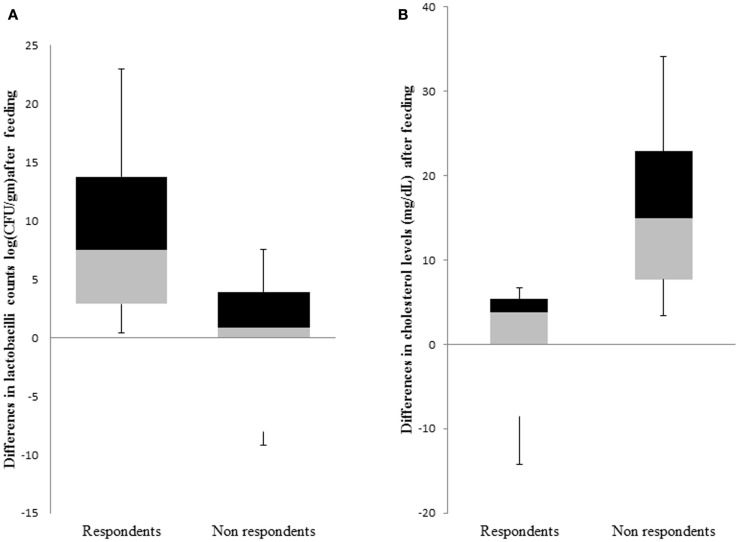
**Comparison of response groups with respect to (A) lactobacilli counts and (B) cholesterol levels**. The middle line represents the median, the box represents the interquartile range, and the whiskers represent the total range.

The primary outcome of this trial was a reduction in TC after 4 weeks of feeding probiotic MTCC 5463. We defined non-respondents as those subjects who experienced elevations in TC of ≥2.509 mg/dL, whereas respondents were the ones who showed no change in TC or <1.72 mg/dL TC in response to the probiotic intervention of 4 weeks. Among the 59 subjects who could complete the study, we classified a total of 16 subjects into respondents (*n* = 8) and non-respondents (*n* = 8) based on cholesterol levels and lactobacilli counts (Figure 2). There were no significant differences in the baseline characteristics of the two groups. This eliminates the influence of gender, weight, and age in influencing the response toward probiotic intervention. The abundance of *L. helveticus* MTCC 5463 was significant (*p* < 0.05) higher in the individuals with an increase in cholesterol levels, as compared to those with a decrease. The decrease in cholesterol levels among respondents was a maximum 14.19% with a 23.66% increase in lactobacilli count in feces. Among non-respondents, a maximum increase of 34.13% in cholesterol with a 9.31% decrease in lactobacilli count was observed. The increase in lactobacilli counts with a decrease in cholesterol in case of respondents indicated that the observed hypocholesterolemic effect of the strain was dependent on the number of lactobacilli in the gut.

### Microbiome diversity estimates associated with respondents and non-respondents

**Figure 3:**
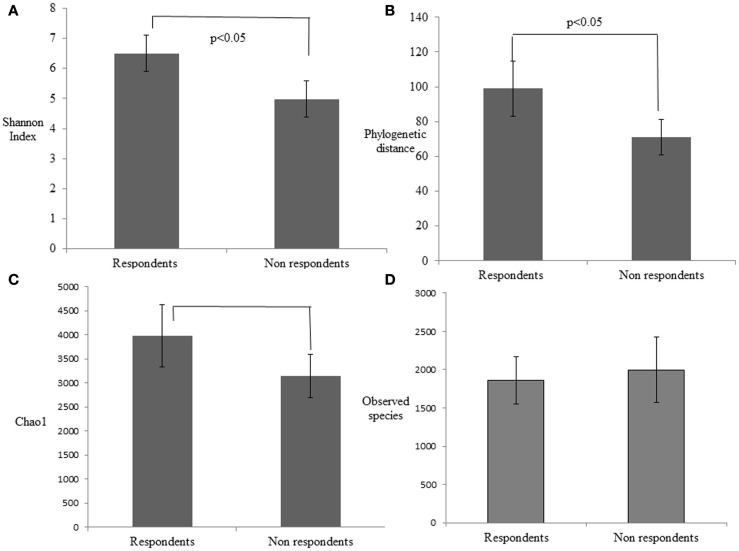
**Estimation of the phylogenetic diversity of the gut microbiota in the respondent and non-respondent groups using the (A) Shannon index, (B) phylogenetic distance, (C) Chao1, and (D) observed species**. The values are means, and error bars indicate the 95% confidence intervals.

The alpha diversity of the respondent group before probiotic feeding (30.8 ± 4.8) and after feeding (26.6 ± 3.6) was higher than non-respondents’ measures for before (25.6 ± 4.5) and after probiotic feeding (24.6 ± 5.8). This indicates that bacterial richness is a factor that promotes responsiveness toward beneficial strains in the gut. To investigate differences in rarefaction measures, we rarified each sample at 33,000 reads and performed the two-sample *t*-test on the two groups. Respondents had significantly greater alpha diversity indices like phylogenetic distance (*p* = 0.022), Chao1 (*p* = 0.019), and Shannon index (*p* = 0.00058) than the non-respondents (Figure 3). A non-significant increase in observed species in case of non-respondents (*p* = 0.27) could be due to presence of distinct commensals that reflect the host’s dietary and geographical differences. There was a non-significant abundance of *Clostridium*, *Shigella*, and *Listeria* among rural respondents. Poor sanitation and hygiene maintained in the rural households could have led to the distinct differences in gut microbiota.

### Bacterial taxa populations associated with respondents and non-respondents

**Figure 4:**
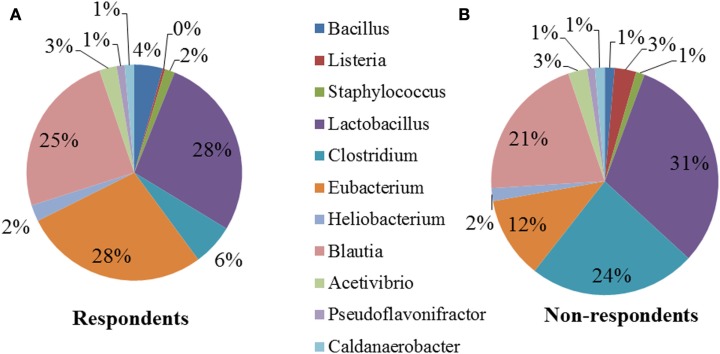
**Relative abundances of the dominant genera (Firmicutes) in (A) respondents and (B) non-respondents**.

**Figure 5:**
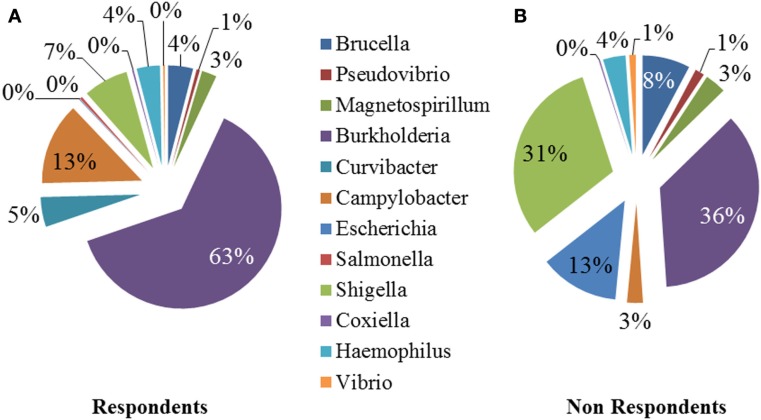
**Relative abundances of the dominant genera (Proteobacteria) in (A) respondents and (B) non-respondents**.

We performed a comparison of the microbiota between respondents and non-respondents to find specialized bacterial members within the abundant phyla *Firmicutes* and *Proteobacteria*. Respondents carried a lower proportion of *Clostridium* and a higher proportion of *Eubacterium* compared to the non-respondents (Figure 4). Surprisingly, although the non-respondents had a higher proportion of gut lactobacilli (31%) compared to 28% in respondents, a favorable reduction in cholesterol corresponding to the increase in strain MTCC 5463 was not observed. This could be due to competitive exclusion by a higher proportion of *Clostridium* (24%) in the gut on non-respondents compared to respondents (6%). The presence of *Listeria* in the non-respondents further emphasizes the need to investigate the association of gut microbiota, especially pathobionts with probiotic strain. Comparing the abundance in the genera of Proteobacteria group, it can be observed (Figure 5) that respondents carried a higher amount of *Burkholderia* (63%) and a lower amount of *Shigella* (7%) compared to non-respondents, who harbored lower count of *Burkholderia* (36%) and a higher amount of *Shigella* (31%), which must have affected the colonization of the probiotic strain. Non-respondents carried a higher amount of *Escherichia* and *Brucella* in the gut. *Shigella* seemed to have a symbiont asymptomatic existence in the host, showing no discomfort to the subjects. The higher amount of *Escherichia* and *Camphylobacter* could be the deciding biomarkers of non-responsiveness toward probiotic intervention.

### Statistical analysis of metagenomic data

**Figure 6:**
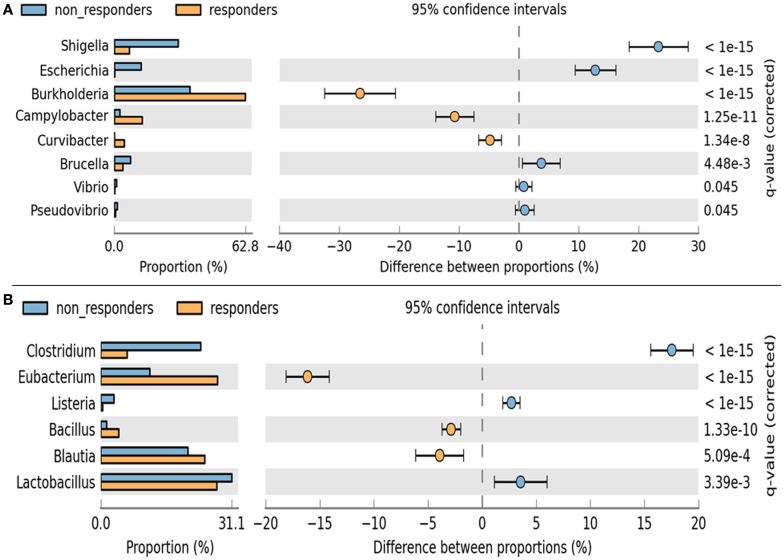
**Analysis of significance in abundance of (A) Firmicutes and (B) Proteobacteria among respondents and non-respondents using two-sample Fischer’s exact test**.

A remarkable significant difference among the chief genera of Proteobacteria including *Shigella*, *Escherichia*, *Burkholderia*, and *Camphylobacter* (*q* < 0.002) was observed. The chief genera of Firmicutes that showed remarkable significant difference were *Lactobacillus*, *Clostridium*, *Eubacterium*, and *Blautia* (*q* < 0.002) (Figure 6). Although non-respondents carried a higher proportion of Lactobacilli, a favorable physiological function may not be translated to the host possibly due to an increase in *Clostidum*, *Shigella*, and *Eschericihia* with a decrease in *Blautia* and *Burkholderia*. We would like to add that the results of population wide samples taken at one time point for a study might not be able to display the entire variation that exists in that population over time and place.

## Discussion

A primary beneficial effect of consuming a bile-salt-hydrolyzing *L. helveticus* MTCC 5463 strain is a reduction in serum cholesterol levels. In the clinical trials carried out to prove the hypocholesterolemic effect of the strain (Ashar and Prajapati, 2001; Prajapati et al., 2012) we could observe participants responding differently to the same treatment. Similar cases of inter-individual variability in response to probiotics (van Baarlen et al., 2011; Grzeskowiak et al., 2012; Arboleya et al., 2013) have been published. Previously, subjects had been classified as respondents and non-respondents based on a greater than or less than 10% change in cholesterol (Cox et al., 2014) but this classification was criticized as being impractical (Ding and Schloss, 2014). Classification using a fecal biomarker (Coen et al., 2009) or biomarkers of the host’s basic metabolism (Naruszewicz et al., 2002; Herron et al., 2003; Ibrahim et al., 2010) has been suggested. We conducted a comparative analysis of fecal microbiomes of respondents and non-respondents to identify bacterial biomarkers. Results of such microbial profiling may serve as a clinically useful biomarker in geriatric care (Kostic et al., 2013).

Primer usage is one of the most critical factors affecting 16S rDNA analysis (Armougom and Raoult, 2009). There exists a possibility that amplification efficacy of the primers could have led to the underestimation of bacterial richness, in this study. We chose the Ion Torrent PGM platform due to its inherent low-cost per sequencing run that allowed us to perform the next-generation sequencing on site at the Veterinary faculty at AAU.

There were no significant differences in the age or weight of respondents and non-respondents, though respondents had a tendency to be older. An effect size of 0.8 among the groups further eliminated the influence of gender, weight, and age toward the expected response. Grouping of similar sequences as operational taxonomic units (OTUs) and quantifying the number of OTUs gave an approximation of species diversity in a sample (Sun et al., 2009; Schloss et al., 2014). Species diversity has been reported as a characteristic feature determining state of health or disease. Reduced colonic microbial diversity of dysbiosis is reported in Crohn disease (Ott et al., 2004), ulcerative colitis (McLaughlin et al., 2010), antibiotic-associated diarrhea (Chang et al., 2008), and *Clostridium difficile* infection (Seto et al., 2014). A higher alpha diversity of gut bacteria among respondents compared to non-respondents seemed to support microbial integrity. Bacterial richness and evenness play an integral role in the success of a probiotic therapy as earlier observed in colitis (Kennedy et al., 2000).

A higher Chao’s estimator and the Shannon index among respondents indicate higher saturation and unevenness in taxa abundance within samples. A non-significant increase in observed species in case of non-respondents suggested the presence of species that may have prevented the probiotic to establish and exert the functionality. We could identify distinct microbial diversity in rural subjects compared to the urban dwellers especially in the presence of *Clostridium*, *Shigella*, and *Listeria*, similar to Russian rural communities (Tyakht et al., 2013). Gram-negative bacteria were more abundant than Gram-positive bacteria in the rural population. *Shigella* and *Escherichia* were significantly under-represented in rural African children than urban European children (De Filippo et al., 2010). In the present study, we observed *Shigella* and *Escherichia* higher in geriatric rural dwellers, which reflected the age and geography-induced diversities in human gut microbiome.

Phyla associated with a healthy state in Indian geriatrics as suggested in this study are Firmicutes accounting for at least 50%, followed by Actinobacteria (20%) and Proteobacteria (10%). At the phylum level, the majority of the intestinal bacteria are known to belong to *Bacteroidetes* and *Firmicutes* (Eckburg et al., 2005). Surprisingly, none of the sequences from the present study were assigned to the Bacteroidetes. An under representation of Bacteroidetes could be due to inter-subject variability (Ley et al., 2006), variation due to adiposity (Frank et al., 2007; Wu et al., 2011), or suppression due to inflammatory bowel disease (Lazarevic et al., 2009). We could not ignore the possibility of loss of this phylum in fecal samples when stored for longer periods and MG-RAST-based classifications’ sensitivity for Proteobacteria (Korpela et al., 2014). Antibiotic usage in elderly can again cause a decline in commensal anaerobes like *Bacteroides*, *Lactobacillus*, and *Bifidobacterium* (Macfarlane, 2014).

Responses of the host to individual bacterial strains are influenced by the baseline composition of the gut microbiota (Rajilić-Stojanović et al., 2015). The respondents showed a lower percentage of Firmicutes and non-respondents showed a comparative lower amount of Proteobacteria. Among Firmicutes, Clostridia were higher in case of non-respondents (24% compared to 6% in respondents). Although the volunteers were not consuming antibiotics during the trial, prior usage of antibiotics could have diminished the population of total and commensal bacteria (Biagi et al., 2010) leading to an overgrowth of *Clostridium* (Round and Mazmanian, 2009) in non-respondents. Antibiotics are readily available over the counter at pharmacies in India and inconsistent hospital standards toward antibiotic usage could have led to higher proportion of Clostridia in the gut. Respondents carried a higher proportion of *Eubacterium* (28% compared to 12% in non-respondents), which reflected a healthy state. An increased diversity of Eubacteria has been observed in the elderly (Hopkins and Macfarlane, 2002). Decrease in *Eubacterium* lead to decreased levels of SCFA, facilitating easier entry of *Enterobacteriaceae* into the intestinal mucosa due to an impaired secretion of mucins by the intestinal epithelial cells (Garrett et al., 2010). The outgrowth of anaerobic *Enterobacteriaceae* must have led to a competitive exclusion of aerotolerant MTCC 55463 strains in the host intestine.

Proteobacteria, recently defined as “pathobionts” (Morgan et al., 2004) are considered to be minor and opportunistic components of the human gut ecosystem. The majority of the sequences assigned to the Proteobacteria were Burkholderiales. A higher proportion of *Burkholderia* is a signature of good health as earlier observed in the healthy Indian child data set (Schultz et al., 2004). Non-respondents had a higher proportion of Proteobacteria, especially *Escherichia*/*Shigella* (indistinguishable as a 16S-based phylotype), previously implicated in intestinal inflammation (Guslandi et al., 2004). Although the volunteers were seemingly healthy with no complaints of gastrointestinal disturbances, a core structural and functional dysbiosis caused by an overgrowth of *Escherichia*/*Shigella* (Malchow, 1997) could have led to a lack of translation of functionality of MTCC 5463 to the host in spite of being present at a higher proportion in the non-respondents gut.

In this study, it was shown that consumption of probiotic yogurt did not significantly reduce TC levels, the intervention significantly reduced serum levels of LDL, TC/HDL ratio, and LDL/HDL ratio in geriatric volunteers. Many studies in literature support the beneficiary effect of probiotics on lipid profiles of subjects. There also exist some contradictory reports where eating probiotic yogurt did not change lipid profiles (Hatakka et al., 2008; Sadrzadeh-Yeganeh et al., 2010). This indicates that apart from the probiotic strain, the host gut microbiome has a big role to play on the response of the host to the probiotic strain.

STAMP analysis revealed proportions of distinct microbial biomarkers like *Shigella*, *Escherichia*, *Burkholderia, Camphylobacter*, *Lactobacillus*, *Clostridium*, *Eubacterium*, and *Blautia* that can help tailor a probiotic therapy to a niche population. The authors would like to strike an analogy to feeding probiotics to a host with imbalanced consortia in the gut to the likes of pouring water to a filled pitcher. Like the water flows out, the probiotic strains are lost in the feces and fail to colonize and translate the functionality to the host. Metatranscriptomic studies could furnish further information for comprehending the molecular basis of responsiveness toward a probiotic therapy because gene expression profiles are more individualized than DNA-level profiles and less variable than microbial composition. Geriatric care is critical in the aging global population and the role of gut metagenomics cannot be overstated in understanding its role in health and disease for the future development of personalized nutrition.

## Conclusion

Globally today, the elderly populations are looking for natural means of sustaining digestive health. Compared to the growing awareness and market penetration of probiotics, there is a dearth of scientific evidence on how probiotics affect the composition of gut microbiota. The well-documented probiotic *L. helveticus* MTCC 5463 was administered to geriatrics in a clinical trial, and a deep sequencing technology was employed to study the changes in the resident microbes over the duration of probiotic consumption. We could find that chiefly *Shigella*, *Escherichia*, *Burkholderia*, *Camphylobacter*, *Lactobacillus*, *Clostridium*, *Eubacterium*, and *Blautia* define the response of the host to the probiotic strain. Moreover, we observed a shift in the gut profile of the non-respondents towards a respondent’s signature gut profile after consuming the probiotic, which proves the importance of precise personalized selection of dosage for an effective tailored probiotic therapy.

## Ethics

Approval for the study design was obtained from the Institutional Ethics Committee (IEC) of Shri Krishna Medical College Karamsad, Anand, Gujarat (HMPCMCE:HREC/FCT/41/01) and Anand Agricultural University (AAU), Anand (AAU/DR/RES/DM/IEC/659/2011). The trial was registered at ICMR Clinical Trial Registry (REF/2012/10/004135).

## Author Contributions

Conceived and designed the experiments: JP, CJ, and HP; performed metagenomic analysis and manuscript writing: SS; product development: SV; clinical recruitment of participants: MG and US; clinical investigations and data interpretation: ST and RP; literature search and critical review of the manuscript: HAP; statistical data analysis: AP.

## Conflict of Interest Statement

The authors declare that the research was conducted in the absence of any commercial or financial relationships that could be construed as a potential conflict of interest.

## Footnotes

^1^http://www.ncbi.nlm.nih.gov/blast/

^2^http://www.ebi.ac.uk/embl/

^3^http://metagenomics.nmpdr.org/

^4^http://kiwi.cs.dal.ca/Software/STAMP

## Abbreviations

μl, microliter; AAU, Anand Agricultural University; CFU, colony forming unit; dL, deciliter; dNTP, deoxynucleotide; ICMR, Indian Council of Medical Research; ISAPP, International Scientific Association for Probiotics and Prebiotics; mg, milligram; ml, milliliter; mM, millimolar; MTCC, microbial type culture collection; nt, nucleotide; PGM, Personal Genome Machine; pmol, picomol; QIIME, Quantitative Insights into Microbial Ecology; rRNA, ribosomal RNA; s, seconds; S, Svedberg unit; U, unit.
